# Toward a global and reproducible science for brain imaging in neurotrauma: the ENIGMA adult moderate/severe traumatic brain injury working group

**DOI:** 10.1007/s11682-020-00313-7

**Published:** 2020-08-14

**Authors:** Alexander Olsen, Talin Babikian, Erin D. Bigler, Karen Caeyenberghs, Virginia Conde, Kristen Dams-O’Connor, Ekaterina Dobryakova, Helen Genova, Jordan Grafman, Asta K. Håberg, Ingrid Heggland, Torgeir Hellstrøm, Cooper B. Hodges, Andrei Irimia, Ruchira M. Jha, Paula K. Johnson, Vassilis E. Koliatsos, Harvey Levin, Lucia M. Li, Hannah M. Lindsey, Abigail Livny, Marianne Løvstad, John Medaglia, David K. Menon, Stefania Mondello, Martin M. Monti, Virginia F.J. Newcombe, Agustin Petroni, Jennie Ponsford, David Sharp, Gershon Spitz, Lars T. Westlye, Paul M. Thompson, Emily L. Dennis, David F. Tate, Elisabeth A. Wilde, Frank G. Hillary

**Affiliations:** 1 Department of Psychology, Norwegian University of Science and Technology, 7491, Trondheim, Norway; 2 Department of Physical Medicine and Rehabilitation, St. Olavs Hospital, Trondheim University Hospital, Trondheim, Norway; 3 Department of Psychiatry and Biobehavioral Sciences, Semel Institute for Neuroscience and Human Behavior, UCLA, Los Angeles, CA USA; 4 UCLA Steve Tisch BrainSPORT Program, Los Angeles, CA USA; 5 Department of Neurology, University of Utah School of Medicine, Salt Lake City, UT USA; 6 Department of Psychology and Neuroscience Center, Brigham Young University, Provo, UT USA; 7 Cognitive Neuroscience Unit, School of Psychology, Deakin University, Burwood, Australia; 8 Department of Rehabilitation Medicine, Icahn School of Medicine at Mount Sinai, New York, NY USA; 9 Department of Neurology, Icahn School of Medicine at Mount Sinai, New York, NY USA; 10 Center for Traumatic Brain Injury, Kessler Foundation, East Hanover, NJ USA; 11 Rutgers New Jersey Medical School, Newark, NJ USA; 12 Cognitive Neuroscience Laboratory, Shirley Ryan AbilityLab, Chicago, IL USA; 13 Department of Physical Medicine & Rehabilitation, Neurology, Department of Psychiatry & Department of Psychology, Cognitive Neurology and Alzheimer’s, Center, Feinberg School of Medicine, Weinberg, Chicago, IL USA; 14 Department of Neuromedicine and Movement Science, Norwegian University of Science and Technology, Trondheim, Norway; 15 Department of Radiology and Nuclear Medicine, St. Olavs Hopsital, Trondheim University Hospital, Trondheim, Norway; 16 Section for Collections and Digital Services, NTNU University Library, Norwegian University of Science and Technology, Trondheim, Norway; 17 Department of Physical Medicine and Rehabilitation, Oslo University Hospital, Oslo, Norway; 18 Department of Psychology, Brigham Young University, Provo, UT USA; 19 George E. Wahlen Veterans Affairs Medical Center, Salt Lake City, UT USA; 20 Leonard Davis School of Gerontology, University of Southern California, Los Angeles, CA USA; 21 Department of Biomedical Engineering, Viterbi School of Engineering, University of Southern California, Los Angeles, CA USA; 22 Departments of Critical Care Medicine, Neurology, Neurological Surgery, University of Pittsburgh, Pittsburgh, PA USA; 23 Safar Center for Resuscitation Research, Pittsburgh, PA USA; 24 Clinical and Translational Science Institute, Pittsburgh, PA USA; 25 Neuroscience Center, Brigham Young University, Provo, UT USA; 26 Departments of Pathology(Neuropathology), Neurology, and Psychiatry and Behavioral Sciences, Johns Hopkins University School of Medicine, Baltimore, MD USA; 27 Neuropsychiatry Program, Sheppard and Enoch Pratt Hospital, Baltimore, MD USA; 28 H. Ben Taub Department of Physical Medicine and Rehabilitation, Baylor College of Medicine, Houston, TX USA; 29 Michael E. DeBakey Veterans Affairs Medical Center, Houston, TX USA; 30 C3NL, Imperial College London, London, UK; 31 UK DRI Centre for Health Care and Technology, Imperial College London, London, UK; 32 Department of Diagnostic Imaging, Sheba Medical Center, Tel-Hashomer, Ramat Gan, Israel; 33 Joseph Sagol Neuroscience Center, Sheba Medical Center, Tel-Hashomer, Ramat Gan, Israel; 34 Sunnaas Rehabilitation Hospital, Nesodden, Norway; 35 Department of Psychology, University of Oslo, Oslo, Norway; 36 Department of Psychology, Drexel University, Philadelphia, PA USA; 37 Department of Neurology, Drexel University, Philadelphia, PA USA; 38 Division of Anaesthesia, University of Cambridge, Cambridge, UK; 39 Department of Biomedical and Dental Sciences and Morphofunctional Imaging, University of Messina, Messina, Italy; 40 Department of Psychology, University of California Los Angeles, Los Angeles, CA USA; 41 Department of Neurosurgery, Brain Injury Research Center (BIRC), UCLA, Los Angeles, CA USA; 42 Department of Computer Science, Faculty of Exact & Natural Sciences, University of Buenos Aires, Buenos Aires, Argentina; 43 National Scientific & Technical Research Council, Institute of Research in Computer Science, Buenos Aires, Argentina; 44 Turner Institute for Brain and Mental Health, School of Psychological Sciences, Monash University, Melbourne, Australia; 45 Monash Epworth Rehabilitation Research Centre, Epworth Healthcare, Melbourne, Australia; 46 Department of Brain Sciences, Imperial College London, London, UK; 47 Care Research & Technology Centre, UK Dementia Research Institute, London, UK; 48 NORMENT, Division of Mental Health and Addiction, Oslo University Hospital, Oslo, Norway; 49 Imaging Genetics Center, Stevens Neuroimaging & Informatics Institute, Keck School of Medicine of USC, Marina del Rey, CA USA; 50 Departments of Neurology, Pediatrics, Psychiatry, Radiology, Engineering, and Ophthalmology, USC, Los Angeles, CA USA; 51 Department of Psychology, Penn State University, University Park, State College, PA USA; 52 Department of Neurology, Hershey Medical Center, State College, PA USA

**Keywords:** Brain injury, Radiology, Open Science, Neurodegeneration, Rehabilitation, ENIGMA

## Abstract

The global burden of mortality and morbidity caused by traumatic brain injury (TBI) is significant, and the heterogeneity of TBI patients and the relatively small sample sizes of most current neuroimaging studies is a major challenge for scientific advances and clinical translation. The ENIGMA (Enhancing NeuroImaging Genetics through Meta-Analysis) Adult moderate/severe TBI (AMS-TBI) working group aims to be a driving force for new discoveries in AMS-TBI by providing researchers world-wide with an effective framework and platform for large-scale cross-border collaboration and data sharing. Based on the principles of transparency, rigor, reproducibility and collaboration, we will facilitate the development and dissemination of multiscale and big data analysis pipelines for harmonized analyses in AMS-TBI using structural and functional neuroimaging in combination with non-imaging biomarkers, genetics, as well as clinical and behavioral measures. Ultimately, we will offer investigators an unprecedented opportunity to test important hypotheses about recovery and morbidity in AMS-TBI by taking advantage of our robust methods for large-scale neuroimaging data analysis. In this consensus statement we outline the working group’s short-term, intermediate, and long-term goals.

## Brain injury and the ENIGMA consortium

For over three decades, neuroimaging has played an important role in the characterization and management of moderate-to-severe traumatic brain injury (msTBI). Novel magnetic resonance imaging (MRI) methods and image analysis techniques have great potential to improve clinical assessment and guide management and treatment following msTBI. For this to be possible, we must first address a number of scientific and practical challenges in our field. The vast heterogeneity of this patient population with respect to injury causes and mechanisms, neuropathology, and clinical or functional outcomes—in combination with the relatively small sample sizes of most current neuroimaging studies—pose significant barriers to scientific progress and clinical translation.

**Fig. 1:**
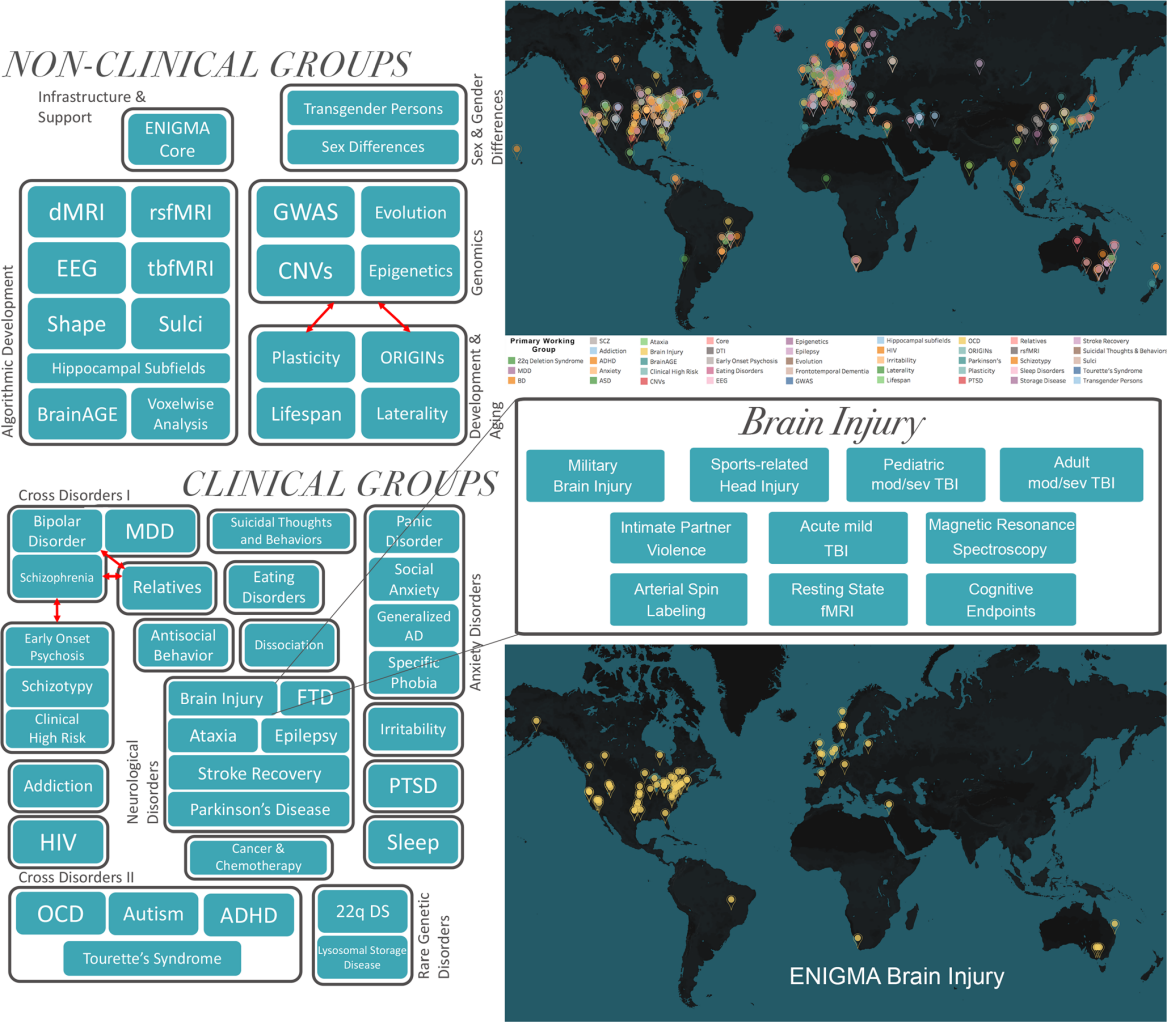
The ENIGMA consortium and the Brain Injury working group. Organization and current geographical representation in the ENIGMA consortium and the ENIGMA Brain Injury working group. Adapted from Thompson et al., 2020 and Wilde et al. 2019

The Enhancing Neuroimaging Genetics through Meta-Analysis (ENIGMA) consortium offers a framework for meta- and mega-analysis of neuroimaging data across sites. This framework has proven to be a successful environment for studying other psychiatric and neurological populations, often with sample sizes 10–30 times larger than those in typical brain imaging studies (Bearden and Thompson 2017; Thompson et al. 2020). In this *consensus statement,* we describe the aims and goals of the ENIGMA Adult^1^ msTBI (AMS-TBI) working group that was initiated in 2018 as part of ENIGMA Brain Injury, which is a collaboration of 10 TBI working groups (Wilde et al. 2019; Fig. 1). The group consists of scientists and clinicians from a wide-range of disciplines and backgrounds, and we welcome new members from around the world to join our efforts.

**Fig. 2:**
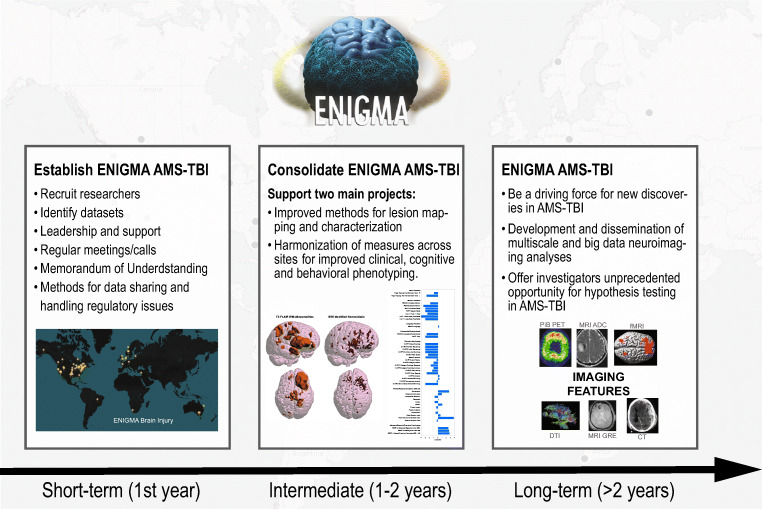
Goals of ENIGMA AMS-TBI. Schematic presentation of the short, intermediate and long-term goals of the ENIGMA AMS-TBI working group

The ENIGMA AMS-TBI working group aims to 1) be a driving force for new discoveries in AMS-TBI by providing researchers with a comprehensive and effective framework and platform for large-scale, cross-border collaboration and data sharing. Moreover, we will 2) facilitate the development and dissemination of multiscale and big data analysis pipelines for harmonized analyses in AMS-TBI using structural and functional MRI in combination with other imaging modalities, non-imaging biomarkers, genetics, as well as clinical and behavioral measures. Ultimately, 3) we will offer investigators an unprecedented opportunity to test important hypotheses about injury neuropathology through recovery and morbidity in msTBI by taking advantage of our robust methods for large-scale data analysis. Below we outline the background and structure of ENIGMA AMS-TBI, the roles of investigators, and the working group’s *short, intermediate, and long-term goals* (Fig. 2).

## Leveraging ENIGMA to address challenges in AMS-TBI research

TBI is a major and increasing global health challenge, with more than 50 million new cases estimated to occur worldwide each year (5 to 20% are msTBI), and an ensuing disability that is 2–3 times higher than the contribution from cerebrovascular disorders or Alzheimer’s disease (GBD 2016 Neurology Collaborators 2019; Maas et al. 2017). TBI is defined as an alteration in brain function, or other evidence of brain pathology, caused by an external force (Menon et al. 2010), including blunt or penetrating trauma, acceleration-deceleration forces or exposure to blast (Thurman and National Center for Injury Prevention and Control (U.S.) 1995).

TBI is currently considered a chronic condition characterized by evolving changes which require precise disease phenotyping, both in the acute stage and during the individual’s lifespan (Corrigan and Hammond 2013; Maas et al. 2017; Masel and DeWitt 2010). Common measures of injury severity, typically based on the Glasgow Coma Scale (GCS) score (Teasdale and Jennett 1974) alone, or in combination with other clinical signs or imaging (e.g. Stein and Spettell 1995), only partially capture the variability in cognitive, behavioral and social outcomes at acute and chronic stages following injury. In the early phase after injury, lower GCS score, older age, pupil dilatation, hypoxia, hypotension, and CT classifications based on the size of lesions and the degree of midline shift, provides some utility for predicting mortality and for categorization of patients into very broad outcome groups (Faried et al. 2018; Maas et al. 2013; Steyerberg et al. 2008). However, this information is less valuable for evaluating patients presenting with less severe injuries or more fine-tuned prognostication of long-term neurobehavioral outcome. Indeed, often individuals with similar indicators of severity and early clinical trajectories experience different outcomes (Bigler et al. 2006; Lutkenhoff et al. 2019). Recovery and community re-integration are further complicated by a number of interacting premorbid, clinical, demographic, and genetic factors (Mollayeva et al. 2019).

An integrated scientific endeavor is required in AMS-TBI research to address these challenges. This will require large sample sizes drawn from a multi-modal approach which combines neuroimaging, non-imaging biomarkers, psychological, cognitive, and behavioral data. Recent developments in neuroimaging and computational algorithms offer powerful approaches to examine not only the overt behavior of individuals, but also the brain structure, function, and neural computations that can give rise to diverse outcomes (Amyot et al. 2015). In addition, there is an unexploited potential of using neuroimaging to inform clinicians about the optimal timing and effects of interventions for individual patients.

In recent years, neuroscientists have encountered problems in the replication of published human neuroimaging studies, especially those based on functional neuroimaging (Poldrack et al. 2017). Many believe that this may be due to small effect sizes; the median statistical power in neuroscience studies has been estimated to be between 8 and 31% (Button et al. 2013). One solution has been to increase sample sizes (Carter et al. 2016; Szucs and Ioannidis 2017). Large-scale collaborative studies are therefore important to move the field of TBI forward (Maas et al. 2017; Tosetti et al. 2013). Standing on the shoulders of successful large-scale initiatives in TBI research such as the TRACK-TBI (https://tracktbi.ucsf.edu/) consortium in the US, the CREACTIVE (http://creactive.marionegri.it/) and CENTER-TBI (www.center-tbi.eu; see Steyerberg et al. 2019) collaborations in Europe, our ENIGMA working group will offer a new platform and framework for researchers to make neuroimaging more useful for understanding AMS-TBI.

**Fig. 3:**
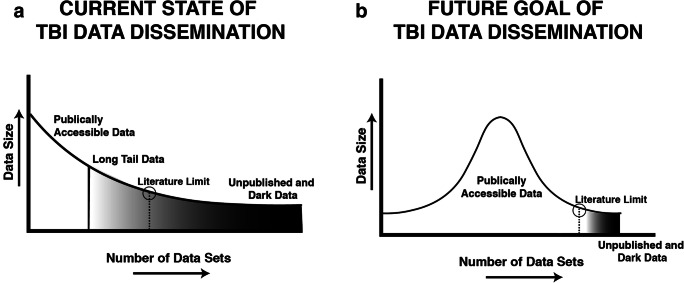
The long tail and dark data for traumatic brain injury (TBI) research. The current state of TBI data consists of a relatively small number of large, publicly accessible datasets reflected schematically as a right-skewed distribution (Panel **a**). The majority of data collected by the field exists in the long tail of the distribution, with most datasets consisting of relatively modest data sizes as either gray data that are difficult to access beyond summaries reported in publications; or dark data that are inaccessible or archived. **b** The goal is to make TBI imaging data Findable, Accessible, Interoperable, and Reusable (FAIR, Wilkinson et al., 2016) thereby shortening the long tail of dark data, and making a greater proportion of the data in the TBI literature publicly accessible to drive new discoveries and accelerate translation. (Adapted from Hawkins et al., 2019)

By offering our framework and methods to the larger research community, we also aim to unlock the enormous potential of analyzing dormant and unpublished “long tail” imaging data (see Fig. 3a,b; Hawkins et al. 2019), which are not being leveraged as part of any coordinated, collective effort (Fig. 3a). Long-tail or “dark data” are data that accumulate in labs after a specific study finding is either interpreted and published through a single study-specific lens or unpublished and then archived. Consequently, there is great potential to integrate and harmonize such data in unique and creative ways. A central goal for our working group is to provide methodological tools to integrate these datasets from TBI labs around the world (Fig. 3b).

In the long term, methodologies developed as part of the ENIGMA AMS-TBI initiative may have broader impact that go beyond research imaging. The lessons learned by integrating data and finding imaging biomarkers with diagnostic, prognostic and therapeutic significance should inform the development of management protocols by clinicians and product development in the biomedical industry. These advances could specifically enhance the use of neuroimaging in the clinical management of TBI through 1) the development and validation of clinically useful methods for analysis that accommodate both high- and low-end imaging protocols (including legacy data), and 2) by informing the development of a future core clinical imaging dataset for TBI, with acquisition parameters and data structure established by broad consensus, that could be harmonized across vendors. Given that the clinical use of MRI dwarfs data acquired for research by several orders of magnitude, such harmonization (which is already occurring in some research contexts, e.g. Alfaro-Almagro et al. 2018; Wiberg et al. 2019) would make very large datasets accessible to research. Collation and integration of such “non-research” clinical imaging for research could deliver analyses that involve datasets with *n* > 100,000. There are clear regulatory barriers and consent hurdles that need to be addressed before such data were freely available for research (Anderson 2015; Benchimol et al. 2015). However, authoritative views suggest that fully anonymized clinical imaging data can be used for research purposes with appropriate safeguards (The Royal College of Radiologists 2017), and such use of data may be further facilitated by federated analyses of data, where the research pipelines are brought to the data (rather than vice versa), both for structural and functional imaging (X. Li et al. 2020; Silva et al. 2019).

## Short-term goal: Forming ENIGMA AMS-TBI—Its structure and methodological framework

Our *short-term goal (1st year)* is to identify datasets and recruit researchers as members by providing an attractive platform and framework for global large-scale cross-border collaboration, data sharing, and analysis. A strength of ENIGMA AMS-TBI is our emphasis on supporting the TBI research community with robust methods and analyses, and the goal to advance brain imaging science in neurotrauma through the principles of *transparency, rigor, reproducibility, and collaboration*.

For ENIGMA AMS-TBI, there is a low threshold for participation (data sharing is not required), allowing individual researchers to choose to participate at different levels depending on their interests and/or situation. There are a number of different ways researchers can participate, these include:1) mega-analyses (sharing *raw data* or numerical output from such data), 2) meta-analyses (no need to share raw data), and 3) methods and protocol development (no need to participate with data). We welcome proposals from the TBI research community at large, and we will serve as a hub for investigators who could benefit from the ENIGMA structure. In addition to working with existing datasets, we will provide a platform for researchers to collect and harmonize future studies. Most data acquired to date have been collected using diverse protocols. Members of the group are developing protocols for future data collection, to enable prospective harmonization within individual cohort studies, thereby allowing members to participate in future multicenter initiatives at *low additional cost and effort*. It would be critically important, in this context, to ensure that we start with what is most universally implementable, and identify a core set of sequences and data collection for widespread use. As with the NINDS Common Data Elements (CDEs; https://www.commondataelements.ninds.nih.gov/Traumatic%20Brain%20Injury#pane-162) it may be useful to also provide more aspirational imaging standards as basic and supplemental - thus allowing optional use of more complex harmonized image collection, if appropriate and possible.

The AMS-TBI working group will benefit greatly from the established procedures, methods, and analytic pipelines that have engendered success across the larger ENIGMA consortium including more than 1400 scientists across 43 countries and more than 20 psychiatric, neurological, and neurodevelopmental disorders (Thompson et al. 2020). Extending prior efforts, we will develop a comprehensive set of protocols, procedures and open source code for data analysis tailored for tackling major challenges in msTBI imaging. Part of this effort can be found in previous ENIGMA programs, which have developed imaging analysis pipelines to extract, homogenize, and control the quality of data describing standardized phenotypes from structural T1-weighted MRI, diffusion MRI, resting state functional MRI and EEG (Adams et al. 2016; Bis et al. 2012; Boedhoe et al. 2017; Guadalupe et al. 2017; Hibar et al. 2016; Hibar et al. 2017, 2015; Hoogman et al. 2017; Ikram et al. 2012; Jahanshad et al. 2013; Schmaal et al. 2016; Stein et al. 2012; van Erp et al. 2016).

One strength of ENIGMA is the focus of researchers *within the consortium* to develop standardized data processing pipelines for handling distinct data types. Much of the variability in research comes from investigators decisions in data processing and analysis, referred to as “researcher degrees of freedom” (see Nichols et al. 2017). In modern neuroimaging, these degrees of freedom can be readily found in analyses of both structural and functional imaging data (Hallquist and Hillary 2019). To standardize approaches for data pre-processing, in particular for functional imaging data pipelines, we plan to integrate members of the international community conducting AMS-TBI work to investigate how to best harmonize and standardize such methods and provide quality control. Overall, the goal for our ENIGMA working group is to act as a forum where AMS-TBI scientists can interact and collaborate, and where consensus on methods can evolve and become suitable for the larger scientific community.

Participating members are encouraged to adhere to the FAIR Data Principles (Findable, Accessible, Interoperable, Reusable), to enhance the usability of data (Wilkinson et al. 2016). Primary data, derived data, and other research outputs such as protocols, source code and software, if well documented, accompanied by descriptive metadata and organized in a standardized way, are likely to foster collaboration and reproducibility. An example of a relevant data repository for publishing data in neuroimaging is OpenNeuro (https://openneuro.org), which uses the Brain Imaging Data Structure (BIDS) format for organizing data (https://bids.neuroimaging.io). By adhering to relevant standards, harmonizing analysis tools and sharing data as open as possible, possibilities for reuse, reproducibility, as well as meta- and mega-analysis greatly increase, both within ENIGMA and in the greater research community.

The ENIGMA AMS-TBI working group will provide support to members on regulatory issues based on accumulated knowledge and available expertise within the network. When combining different data for analysis, there are many levels of sharing, ranging from sharing the raw data, to sharing quantitative measures and features extracted from imaging scans, to sharing only meta-data. It is important to consider the type of data to be shared and the local (institutional, national or international) rules and regulations that need to be followed. There is, therefore, not a single approach, and each participating site needs to abide by appropriate regulations. Our members have extensive experience in dealing with such issues, not only from participation in other ENIGMA groups, but also through participation in other large-scale international TBI collaborations (e.g., CENTER-TBI).

Working group chairs provide leadership to support researchers in achieving planned objectives. Our approach is based on the principles of team science and our success is expected to be driven by the collective coordinated effort of participating researchers. Building on years of experience from ENIGMA, we have developed a group-specific memorandum of understanding (MOU), with policies for data sharing, authorship, and for initiating new studies. Communication within the group will largely involve teleconferences with alternating scheduling to accommodate members across different time-zones, in addition to face-to-face meetings, often in connection with international conferences.

## Intermediate goal: Provide tools for improved lesion mapping and clinical, cognitive and behavioral phenotyping in AMS-TBI

Our *intermediate goal (1–2 years)* is to support two overarching projects to address key challenges linked to the heterogeneity of msTBI which will benefit all future ENIGMA AMS-TBI projects. The first project will focus on developing improved methods for lesion characterization, mapping, and quantification. The second project will focus on harmonization of measures across sites to allow for improved clinical, cognitive, and behavioral phenotyping. This will provide the research community with important methods to directly address two of the main challenges regarding clinicopathological heterogeneity in msTBI. These projects will also serve as important vehicles to motivate researchers to join our early efforts, and for consolidating our working group.

### Standardization of image analysis protocols for AMS-TBI: Improved methods for lesion characterization, mapping, and quantification

**Fig. 4:**
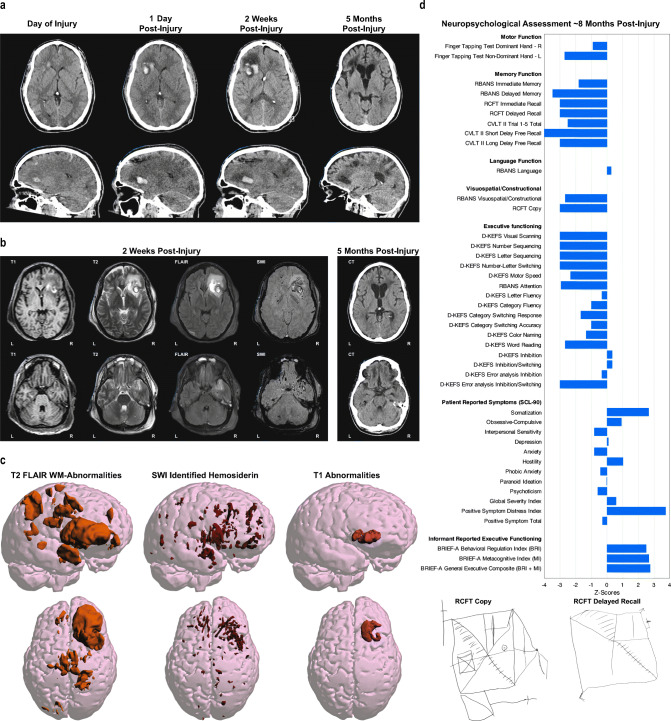
The complexity of lesion characterization and behavioral phenotyping after AMS-TBI. From a structural neuroimaging perspective trauma-induced abnormalities differ by time post-injury as well as the imaging modality being used. **a** are all CT based showing that the size and location of the hemorrhage, parenchymal displacement and edema dynamically change over time. **b** demonstrates that each MRI sequence has its own unique sensitivity in assessing different aspects of neuroanatomy and neuropathology. **c** which presents the FLAIR, SWI and T1 signal abnormalities, demonstrates the widespread pathology differently presented by these imaging methods. By 5 months’ post-injury, widespread volume loss, cortical atrophy, ventriculomegaly and encephalomalacia have occurred. **d** show summary findings from a neuropsychological assessment at ~8 months post injury. This case example depicts the neuropathological heterogeneity associated with TBI along with the dynamic changes over time and their influence on neuropsychological test results. This patient sustained a severe TBI from a motorcycle collision with a vehicle. The patient was not helmeted at the time of injury and, by witness accounts, was immediately rendered unconscious. Upon emergent care at the scene of the accident, the patient was assessed to have a Glasgow Coma Scale (GCS) of 3, was life-flighted to a Level I emergency department (ED) with GCS remaining 3 throughout transport and during ED assessment and treatment. In addition to the head injuries he sustained multiple systemic injuries including leg and rib fractures, pulmonary contusion and liver laceration. An intracranial pressure monitor was inserted, the patient underwent tracheostomy for airway management and shunted. The patient remained in a coma and received neurocritical care for almost 2 months, followed by 3 months of inpatient neurorehabilitation. **a** Initial day-of-injury computed tomography was performed about 90 min’ post-injury. What is important to note in the initial scan is the original size of the frontal intraparenchymal hemorrhage along with the size, symmetry and configuration of the ventricular system. Within 24 h, enlargement of the intraparenchymal hemorrhage is observed along with distinct effacement of the anterior horn of the lateral ventricle and surrounding edema associated with the hemorrhage. Subsequent to this scan he was shunted, with the shunt catheter clearly visible in the 2-week follow-up scan which depicts more edema and midline shift. By 5 months’ post-injury, there is prominence of the ventricular system and cortical sulci in association with cortical atrophy and frontal encephalomalacia associated with the location of the prior hemorrhage. **b** At 2 weeks post-injury, MRI studies were obtained. Each sequence demonstrates a different aspect of the “Lesion.” The T1 sequence, which is the one commonly used for automated methods of image segmentation and classification for quantitative analyses, depicts coarse anatomical features of the brain, but the focal intraparenchymal hemorrhage and surrounding edema is not fully appreciated, being better distinguished by the T2 and FLAIR sequences. The SWI sequence depicts multiple, bilaterally scattered foci of hemosiderin deposition reflective of shear injury, with particularly exquisite demarcation differentiating hemorrhage, parenchymal degradation along with the surrounding edema. **c** Using a thresholding method for detecting white matter signal abnormality in FLAIR scans, the regions of white matter hyperintensity are depicted three dimensionally in the images on the left. Each signal abnormality likely reflects localized white matter pathology. In the middle are the regions of hemosiderin deposition detected on SWI, likewise reflecting specific foci of shear-lesion pathology constituting diffuse axonal injury. On the right are the abnormalities found on T1. **d** Findings from neuropsychological assessment at almost 8 months post injury are presented as z-score deviations from test manual normative data. The following tests were administered: Repeatable Battery for the Assessment of Neuropsychological Status (RBANS, https://www.pearsonassessments.com/), Rey Complex Figure Test (RCFT, https://www.parinc.com), California Verbal Learning Test-II (CVLT-II, https://www.pearsonassessments.com/), Delis-Kaplan Executive Function System (D-KFES, https://www.pearsonassessments.com/); Symptom Checklist-90 (SCL-90, https://www.pearsonassessments.com/) and the Behavioral Rating Inventory of Executive Function (BRIEF, https://www.parinc.com). Clinically, the 25-year-old presented with left side hemiparesis, emotional lability and major cognitive impairments, most notable in terms of memory and executive functioning. Family and caregivers were most concerned about the patient’s irritability and inappropriate outbursts along with impaired insight and judgment. Neuropsychological tests (lower z-scores = poorer function) demonstrated the expected left side reductions in motor control (reduced finger tapping and grip strength) consistent with the location of the large intraparenchymal right frontal hemorrhagic injury (see Fig. 4a-c). He was anosmic and unable to identify basic odors on the Smell Identification Test (https://sensonics.com/) along with diminished tactile discrimination on the left side, but no visual field defect. Constructional praxis was diminished as evident in the copy of the Rey Complex Figure Test (RCFT), with the more profound deficits most notable with impaired immediate as well as delayed memory. Memory and executive impairments were evident on the RBANS, CVLT-II and DKFES tasks. Caregiver observation, based on the BRIEF (higher z-scores = more problems) also confirmed real-world deficits in day-to-day impairments in planning, organization, decision making and problem solving. Emotionally, as also reflected in the BRIEF results, the family caregiver reported marked dysfunction in emotional regulation with poor self-monitoring and impaired insight. In contrast, on the SCL-90 (higher z-scores = more symptoms), which is a self-report measure, while somatic issues that related to mobility and pain were prominently endorsed, the Global Severity Index (GSI) was only minimally elevated, with no significant endorsement of symptoms related to depression or anxiety. This would be consistent with caregiver observations that the patient lacked insight into changes in personality and emotional control, impairments often reported to be present in TBI patients with extensive frontotemporal pathology (Krudop & Pijnenburg, 2015), as evident in this patient

Our working group will aim to provide standardized best practices (e.g. Nichols et al. 2017) for multimodal neuroimaging analysis in AMS-TBI. This need is critical partly because analyzing MRI scans from AMS-TBI patients poses unique challenges from the standpoint of lesion mapping, pathology characterization, and clinical interpretation (see Fig. 4). The heterogeneity of lesion profiles (e.g, biomechanical cause, type of pathology, location, or volume) frequently makes automatic MRI analysis pipelines break down or fail due to causes that frequently include (but are not exclusive to) inaccurate co-registration of scans across modalities and time points, faulty voxel-wise morphometric analysis, and incorrect automatic parcellations of brain structures (Irimia et al. 2014).

Our working group will aim to propose, implement and validate standards to facilitate such operations and to enhance their reproducibility. Even relatively “simple” image processing steps like “skull-stripping” - which is required for many processing pipelines and brain co-registration - tend to fail when using conventional software on images from patients with TBI, and require customized pipelines (Lutkenhoff et al. 2014). Conventional lesion mapping approaches - such as pathology masking - can fail, especially when multiple large lesions are present (Wong et al. 2016). This is partly because masks frequently classify voxels from different lesion types identically regardless of their presentation on MRI, and discard potentially valuable information on lesion type and location, factors that may have prognostic utility (B. Wang et al. 2013). Furthermore, lesion masks do not convey either the pattern or the extent of injury-related brain deformations. As a result, careful testing and validation—including visual inspection by neuroradiologists—can be necessary even when masking techniques have been validated on systematic lesioning data sets. There has been growing interest in using machine learning (ML) to improve anatomical parcellation (Ledig et al. 2015) and lesion detection based on computed tomography (CT) (Jain et al. 2019) and anatomical MRI (Kamnitsas et al. 2017) in AMS-TBI patients. Combining such methods with large databases of systematic lesions (Wang et al. 2013) may be particularly advantageous for connectome analysis (Irimia and Van Horn 2014) or when the alternative involves laborious manual delineation. One aim of our working group will be to propose detailed procedures to integrate information from different sources and methods and to provide guidelines on their use.

As an example, we aim to provide distinct lesion mapping decision trees that accommodate the availability—or partial lack—of MRI scans acquired using various sequences, including *T*_*1*_-weighted (*T*_*1*_w), susceptibility weighted imaging (SWI), and fluid-attenuated inversion recovery (FLAIR) scans. TBI lesion characterization is a complex inferential process, which aims to identify the lesion’s content, physical properties and evolution based on complementary information from a variety of MRI modalities. After image preprocessing, distinct MRI modalities can be used to extract unique information on the physical content, pathophysiological state or likely longitudinal trajectory of each lesion-confined voxel (Wang et al. 2013). For example, the *T*_*1*_w MRI contrast is indicative of the content of fat, whereas FLAIR hyperintensities are linked to the localized tissue water content (e.g. suggesting vasogenic edema or perivascular CSF). By contrast, SWI hypointensities results from the presence of (*i*) ferromagnetic hemoglobin in the lumina of blood vessels and (*ii*) extravasated ferromagnetic material in the cerebral parenchyma. Consequently, lesion description can be challenging in AMS-TBI because inferring MRI signal provenance does not equate straightforwardly to the characterization of pathobiology. For this reason, when lesion-related information is made available from fewer—rather than more—information channels, subtle yet consequential issues of interpretability and diagnosis may arise. To address such difficulties, our working group will aim to formulate a detailed protocol and implement conservative guidelines for lesion mapping, quantification and characterization based on the rigorous understanding and interpretation of the available MRI sequence modalities and on their correct joint interpretation.

### Harmonizing protocols for improved clinical, cognitive, and behavioral phenotyping through large-scale datasets

Accurate patient diagnosis and prognostication, with respect to clinical, cognitive, and behavioral outcomes, is paramount within the TBI field. A sound clinical evaluation of an individual patient includes information about premorbid factors, injury-related variables, and broad clinical and functional assessments that are integrated and appropriately interpreted or formulated (see Fig. 4). In the scientific literature, only general associations have emerged between premorbid, clinical, and demographic factors and subsequent outcomes (Ponsford et al. 2008; Spitz et al. 2012; Wood and Rutterford 2006). For example, history of emotional disturbance, older age, and higher severity of injury generally lead to poorer functional outcomes (Hoofien et al. 2001; Spitz et al. 2019). More recently, there has been significant investment in identifying reliable biomarkers to aid in the initial diagnosis and characterization of TBI and prediction of future outcomes, ultimately, to enable tailored clinical interventions (“Precision Medicine”). This quest has included physiological and neuroimaging measures.

Group-level results suggest that anatomical and functional alterations to the brain generally correlate with changes in cognition and behavior (e.g. Bonnelle et al. 2012; Brezova et al. 2014; Håberg et al. 2015; Kinnunen et al. 2011; Olsen et al. 2015). Brain changes have been characterized with respect to loss in regional volume, altered white-matter microstructure, functional connectivity and brain activation. Despite the application of advanced neuroimaging techniques to TBI, including diffusion-weighted imaging and functional connectivity analyses that can reveal subtle brain changes, the vast majority of the variability in outcomes remains unexplained. This situation clearly highlights the problem of heterogeneity in TBI outcomes and raises the need for ENIGMA-type large-scale research projects. The lack of reliable predictive biomarkers hamper the development of disease-modifying therapies. Moreover, there is difficulty in translating results obtained at the group-level to the individual, likely due to large variability in regard to patient preinjury/genetic profile, demographics, injury mechanism, type and location and post-injury interval/phase (Fisher et al. 2018; Moen et al. 2016; Molenaar et al. 2009). Failure to deduce facts from groups to individuals is probably a major factor explaining the failure of therapeutic interventions (L. M. Li et al. 2014; Saatman et al. 2008). Therefore, accurate individual-specific diagnosis must precede the development of effective treatments.

The problem of heterogeneity is not unique to TBI. Many other fields—for example, psychiatry—also face the ‘heterogeneity problem’ (Feczko et al. 2019); 1) that any outcome or constellation of symptoms is not caused by a single mechanism, but is the result of variable combinations of known and unknown factors; and 2) that our way of measuring individual outcomes influences how we determine the relevant contribution of the potential mechanisms. For example, MRI measures that best *diagnose* TBI may differ from those that best *predict* development of emotional disturbance, or manifestation of any other behavior, following TBI. Defining adequate, clinically relevant, and agreed-upon outcome measures poses a serious challenge. The interagency Traumatic Brain Injury Outcomes Workgroup addressed primarily clinical research objectives (Hicks et al. 2013; Wilde et al. 2010). The rationale behind the core measures was the need to create a primary set of well-established measures that address outcome domains in many studies. This group sought to identify a single measure or limited set of measures that best represented each domain. One of the primary objectives was to facilitate comparability of outcome measurements across studies. Important efforts such as the Common Data Elements (CDE) initiative have provided some direction for researchers for selecting CDEs linked to demographics, acute clinical assessment, neuroimaging, biomarkers/specimens and outcome measures (Duhaime et al. 2010; Thurmond et al. 2010; Yue et al. 2013). Also, the Traumatic Brain Injury Endpoints Development (TED) Initiative aims to provide harmonization of study measures across eight major TBI studies (Manley et al. 2017). However, most existing msTBI studies do not adhere to the CDEs or other standards. ENIGMA AMS-TBI will work with existing initiatives focusing on prospective or retrospective harmonization of measures and data across studies with an aim to contribute to a global solution to this challenge.

One avenue for tackling the challenge of heterogeneity is by leveraging large-scale collaborative initiatives. The ENIGMA AMS-TBI working group offers: a) the ability to standardize quality assurance (QA) and imaging protocols across sites; b) the potential for harmonization of current and future demographic, clinical, and behavioral measures across sites. This will be accomplished by finding CDEs across cohorts—what measures have most commonly been collected and offer the most overlap across sites. Incorporated into this pipeline will be methods that handle, compare, and impute missing information from existing data; c) an open discussion forum to establish a consensus regarding relevant and appropriate measures for diagnosis as well as prognosis within msTBI. The inclusion of clinicians and clinical researchers in the ENIGMA AMS-TBI initiative will contribute to sound discussions of what behavioral, cognitive and other psychological outcome measures are most likely to provide the most relevant optimal benchmark for imaging data; and d) given the larger sample size, the ability to begin using new tools and techniques to better examine clinical, cognitive, and behavioral phenotypes or subgroups of patients. Advances in computational and machine learning approaches may hold a transformative potential to more accurate patient classification opening avenues toward a more *personalized medicine* in AMS-TBI. Our ENIGMA AMS-TBI initiative will facilitate these goals (a–d) and even allow for collection of new data as a consortium to fill gaps or deepen phenotyping.

## Long-term goal: ENIGMA as a sustainable and driving force for new discovery in AMS-TBI

Our *long-term goal (>2 years)* is to be fully engaged with the broader TBI research community and support researchers in tackling important research questions in AMS-TBI, focusing on the unique contributions of big data approaches. We expect the ENIGMA strategy to be ideally suited to particular research questions and our early efforts will leverage our primary strengths of data sharing and methods development. Here, we outline a number of areas where we have current expertise within ENIGMA AMS-TBI, and where we believe our approach has a lot of potential for high gains in the field. Examples are provided recognizing that our group is in its early phase, anticipating that the approaches and initiatives will be shaped further by existing and new members.

### Conducting international replication/reproducibility effort in AMS-TBI

With the replication crisis that emerged in the social sciences in 2015 (Maxwell et al. 2015; Open Science Collaboration 2015) and expanded to nearly every corner of science, including the neurosciences (Button et al. 2013) there have been recent efforts to galvanize the community around specific processing pipelines (see Esteban et al. 2019). In concert with these efforts we aim to work with the international community to leverage the power of data sharing in order to identify the most robust findings in the TBI literature.

To do so, the ENIGMA AMS-TBI aims to establish reliable findings in the imaging and genetics community that can serve as anchors to the field. From these vantage points, the science of TBI can then advance on a firmer scientific footing. Given the range of possible premorbid and injury-related factors that influence the central neural system (CNS) and its functions (behavior), there remain great challenges in the study of reproducibility in TBI research. The promise this effort holds, however, is to determine if key findings emerging from the imaging literature are generalizable across sites and samples, thus providing investigators with a foundation from which they can work. Establishing those reliable findings is vital for the advancement of our understanding of the consequences of TBI on neural systems and patient outcomes. The ENIGMA AMS-TBI working group will vet the first generation of replication studies with the TBI community and begin designing analyses based upon data currently existing amongst our collaborators. Moreover, we invite investigators in the TBI research community to propose critical topics that require replication and can be supported by the ENIGMA AMS-TBI working group. Establishing the reproducibility of our science is a core agenda item for the ENIGMA AMS-TBI working group.

### Acute/early MRI for guiding intervention and prognosis

While CT imaging will continue to play an important role in clinical decision-making in the acute treatment of AMS-TBI (Irimia et al. 2019), increased attention has been given to the clinical and prognostic value of acute/early MRI. Although the optimal timing of MRI acquisition after AMS-TBI is still unknown and may be both injury-specific and patient-specific, imaging does need to be performed early enough to inform clinical decision making. Taking an acute patient for an MRI scan from an intensive care unit (ICU) while under ventilation can be challenging, but remains a vital means for assessment when precautions are taken to ensure MRI compatibility and safety (Carter et al. 2013; Newcombe et al. 2008; Newcombe and Menon 2016).

Early MRI has been successfully implemented to assess the presence and evolution of brain lesions due to trauma (Newcombe et al. 2016, 2013). For example, the presence of brainstem lesions has been linked to increased mortality and unfavorable Glasgow Outcome Scale at 6 months (risk ratio, 1.78; 95% CI, 1.01–3.15; I = 43%) (Haghbayan et al. 2017), while lesions involving the ascending arousal network may be critically predictive of poor outcome (Izzy et al. 2017; Moe et al. 2018). However, the accuracy and replicability of such findings will benefit from the analysis of larger samples from multiple sites. Additionally, greater exploration of the functional impact of injury to additional brain regions and the manner in which the same regions are impacted across imaging modalities is needed.

In addition to prognosis, acute/early MRI may provide key information on the pathophysiological processes of specific lesion types. Contusions in TBI tend to have distinct regions: a core of restricted diffusion associated with hematoma, surrounded by an area of raised apparent diffusion coefficient (ADC) likely to be due to vasogenic edema, and in earlier scans (within 72 h) an outer rim of ADC hypointensity that is later subsumed by the vasogenic edema (Newcombe et al. 2013). This outer rim may represent a region of microvascular failure resulting in cytotoxic edema, and may represent a “traumatic penumbra” which may be rescued with effective therapy. Indeed, in such “at-risk” regions of metabolically compromised tissue, normobaric hyperoxia has been shown to increase oxygen utilization using ^15^O PET and, thus, may help save the metabolically compromised tissue (Nortje et al. 2008). This is consistent with a subsequent study which found that normobaric hyperoxia may pseudo-normalize the ADC in the cytotoxic rim (Veenith et al. 2014). Acute and early clinical MRI, in conjunction with carefully executed experimental animal studies, can also shed light on the mechanisms underlying msTBI (Lutkenhoff et al. 2019). Moreover, the value of early MRI is not limited to structural scans. For example, a functional MRI study in patients with post-traumatic amnesia found evidence of disconnection between the medial temporal lobes and the default mode network (De Simoni et al. 2016). ENIGMA AMS-TBI will work on improved methods to delineate the optimal timing of MRI after AMS-TBI and to further identify and refine lesion patterns yielding important prognostic information which can guide clinical decision-making.

### Imaging disorders of consciousness (DOC) after TBI

Progress in intensive care medicine has led to a large increase in the proportion of patients who survive msTBI (Laureys and Boly 2008; Masel and DeWitt 2010). A majority of AMS-TBI survivors enter a transient state of coma, which is generally considered to resolve within 3 to 4 weeks (Young 2009), to then spontaneously regain the two cardinal elements of consciousness: arousal and (self-)awareness (Laureys 2005). Conventional structural MRI, DTI, and fMRI can provide added prognostic accuracy to the clinical observations and CT imaging in predicting which patients will emerge from coma (Snider et al. 2019; Stevens et al. 2014). A small number of patients with very severe TBI (Beaumont and Kenealy 2005; Løvstad et al. 2014; van Erp et al. 2015), however, fail to fully regain consciousness and enter (transiently or for prolonged and sometimes life-long periods) into a vegetative (VS) or a minimally conscious state (MCS) (cf., Giacino et al. 2002; Jennett and Plum 1972; Monti et al. 2010a). In the context of these three conditions (i.e., coma, VS, MCS) - often referred to as Disorders of Consciousness (DOC) - diagnosis and prognosis are a critical challenge (Monti et al. 2009; Owen and Coleman 2008). In the absence of an objective means of determining level of consciousness, differentiating an MCS from a VS is an inferential process (Giacino et al. 2014) which is known to be logically problematic (cf., Monti and Owen 2010) and prone to misdiagnosis (Schnakers et al. 2006, 2009). However, accurate diagnosis of DOC is essential for medical management, prognosis, monitoring of interventions, as well as the complex legal and ethical ramifications concerning end-of-life decisions. Over the last 20 years, neuroimaging has revolutionized our understanding of these conditions (Lutkenhoff and Monti 2016). Functional MRI has shown the ability to detect both residual cortical processing and networks (e.g., Laureys et al. 2000; Menon et al. 1998; Monti, Pickard, and Owen 2013; Owen et al. 2005) and voluntary (brain) behavior (e.g., Bardin et al. 2011; Edlow et al. 2017; Monti et al. 2015; Monti et al. 2010b) in a minority sub-group of otherwise unresponsive patients. ^18^F-FDG-PET has been shown, in a recent clinical validation study (Stender et al. 2014), to be able to detect the presence of awareness in DOC with greater sensitivity than fMRI (93%) and to predict long-term outcome with high accuracy (74%).

While traditional readings of structural imaging data (e.g., CT, MRI) have shown limited utility in DOC, more advanced analytical and imaging techniques yield greater promise in their ability to uncover patterns of damage in large-scale brain networks (Monti 2012; Schiff 2010), considered hallmarks of DOC, and to differentiate between diagnostic categories. Advanced (i.e., “shape”) analysis of routine T1-weighted data, for example, has demonstrated a link between thalamic and extra-thalamic subcortical atrophy and depth of impairment in chronic DOC patients across etiologies (Lutkenhoff et al. 2015) - a pattern of atrophy which, at least in TBI, might take shape in the first months post injury (Lutkenhoff et al. 2019; Schnakers et al. 2019). Diffusion MRI can also help in quantifying the structural integrity of white matter, and thus potentially the primary and secondary network damage encountered in DOC (Voss et al. 2006). Several recent studies suggest that DTI-derived metrics of fractional anisotropy and diffusivity may be useful in differential diagnosis through the identification of the neural networks underlying the various levels of impairment seen in DOC (Wu et al. 2016; Xu et al. 2017; Zheng et al. 2017). Nonetheless, several gaps in the literature and challenges in applying neuroimaging techniques to DOC still remain (Cavaliere et al. 2014). Patients with prolonged DOC are relatively few, and imaging these patients is challenging. Most imaging group studies of DOC are performed on patients with mixed etiology (e.g., anoxia, stroke), despite known differences across etiologies (Adams et al. 2000; Adams et al. 1999; Giacino and Kalmar 1997; Graham et al. 2005; Lutkenhoff et al. 2015; Multi-Society Task Force on PVS 1994). Our working group will provide a platform to combine data from DOC patient groups across sites and develop improved methods for using imaging in diagnosis and outcome predictions which will be of great value for patients and their caregivers.

### Imaging in treatment and rehabilitation after msTBI

Most intervention studies report results at the group average level, rendering little information on who might benefit from a rehabilitation protocol or what might be the structural or functional underpinnings of treatment efficacy. There is, however, a growing literature using neuroimaging methods to assess system-level plasticity as a result of specific rehabilitation protocols (for a critical review, see Caeyenberghs et al. 2018), including efforts to develop biomarkers for motor (Lima et al. 2011) and cognitive (e.g. Arnemann et al. 2015; Chen et al. 2011; Chiaravalloti et al. 2015) change. Moreover, using neuroimaging as decision aids in stratifying treatment response and supporting treatment selection has a great potential. As an example, a recent study combining MRI and I-ioflupane SPECT demonstrated that only TBI patients with low caudate dopamine transporter levels had cognitive improvements from methylphenidate treatment (Jenkins et al. 2019).

There is a need for large, well-controlled studies that include neuroimaging data to better understand the neural underpinnings of treatment efficacy and individual injury-related factors (Vander Linden et al. 2018) that contribute to success or failure of a given intervention. One goal of ENIGMA AMS-TBI is to support analyses of effects of interventions on broad cognitive processes even in the context of distinct imaging and rehabilitation protocols. This approach will seek to isolate the most robust main effects irrespective of between-study differences, which may guide more nuanced work to examine mechanisms. Currently, the only method to examine main effects is to perform meta-analysis work limited to combining studies by cognitive modality (e.g., interventions that aim at improving memory or attention). However, such an approach is still restricted by the absence of harmonization in scanning protocols and outcome variables, potentially calling for the use of data reduction techniques (such as exploratory principal component analysis on disparate neuropsychological data) and use of multiple covariates (sample size permitting). Second, to understand the efficacy of distinct rehabilitation protocols across a range of behaviors (e.g., improvements in attention and/or memory), we will facilitate prospective work by supporting data harmonization and analyses. While there remain important challenges, data sharing offers the opportunity to orient a community of researchers around common goals of understanding how to ideally study neuroplasticity in the context of neurorehabilitation; it will be a goal of our working group to advance these efforts.

### Testing specific hypotheses about functional brain plasticity after AMS-TBI

Through the use of functional brain imaging approaches (typically fMRI), investigators are frequently interested in the basis of brain plasticity, commonly referred to as neural “reorganization”, following AMS-TBI. Reorganization is often loosely applied to refer to the broad class of anatomical structural and functional alterations in the human brain when performing behavioral tasks after TBI. For some changes post-AMS-TBI, terms such as “compensation” are often used interchangeably with reorganization which has led to confusion and even controversy (see Hillary 2011; Turner et al. 2011). However, it is possible to define a priori hypotheses that predict what functional changes are associated with which theories of anatomical and functional remodeling in the brain (Hillary 2008; Hillary et al. 2006; Medaglia 2017; Medaglia et al. 2012; Olsen et al. 2015; Turkeltaub, *in press*; Venkatesan and Hillary 2019) and even integrate alternative methods including EEG and DTI to examine mechanisms of increased frontal activation commonly observed in TBI (see Olsen et al. 2020). It is further important to use brain-behavior analyses that distinguish competing theories of dysfunction from those of adaptive neuroplasticity. What is required is additional power to examine distinct clinical subgroupings and how systems-level plasticity alters behavioral outcome. ENIGMA will provide a platform to support well-powered studies that refine and systematically examine brain reorganization hypotheses in AMS-TBI.

With the emergence of network neuroscience, analyses of functional MRI data now often include network analyses and a number of studies have focused on how msTBI alters distributed neural systems using graph theory and other approaches (for a review, see Caeyenberghs et al. 2017). Several useful heuristics have emerged from this literature including the observation that TBI may result in enhanced connectivity, or hyperconnectivity (see for a review Hillary and Grafman 2017; Hillary et al. 2015) but this is juxtaposed to other studies documenting disruption in large-scale networks, including the default mode network and salience network as fundamental to problems with set-shifting and attention (see Bonnelle et al. 2012, 2011; Jilka et al. 2014). Thus, while hyperconnectivity has been demonstrated during recovery (<1 year post injury; Bernier et al. 2017; Hillary et al. 2014; Nakamura et al. 2009) and in chronic TBI subjects (see Palacios et al. 2012; Sharp et al. 2011; Venkatesan et al. 2015), the finding is clearly not universal (see Sharp et al. 2014). Clarification is needed, and can be achieved by leveraging large samples that are well-defined with regard to age at injury, time-post injury, and other clinical indicators; such scale and detail may facilitate the exploration of the circumstances where hyperconnectivity is present and its possible associations with clinical outcome.

### Chronic msTBI, aging and risk for neurodegeneration

Efforts are currently underway within the working group for understanding the chronic and long-term effects of TBI on patient functioning. This area of work has gained significant attention over the past decade as there has been increased interest in understanding TBI as a chronic health condition (Corrigan and Hammond 2013). The link between TBI and dementia is supported by some large cohort studies (Guo et al. 2000; Plassman et al. 2000) including the demonstration of a dose-response relationship between injury severity and increased risk of Alzheimer’s disease and dementia. Other studies have not found this association (Weiner et al. 2017). However, there is evidence for broader links between TBI and neurodegeneration, including microinfarcts, synucleinopathies and Parkinson’s disease (Crane et al. 2016; Dams-O’Connor et al. 2016). Multiple mechanisms have been suggested to explain how TBI may be linked to neurodegeneration, including impaired immune function/inflammation (Jassam et al. 2017; Wagner and Kumar 2019) increased vascular risk which has strong links to neurodegeneration (Sweeney et al. 2018; Zlokovic 2011) and alterations in large-scale neural networks leading to disconnection (Hillary and Grafman 2017; Jones et al. 2016). In vivo neuroimaging tools can contribute substantially to the understanding of mechanistic links between TBI and neurodegeneration. There is a critical need to examine processes that reflect the phenomenon of *“aging-with-TBI”* and addressing this issue has become a priority in the study of TBI (see National Institute of Neurological Disorders and Stroke 2018). Considerable investments have been made in characterizing the pathological sequelae of repetitive “subconcussive” head trauma (Hirad et al. 2019; McKee et al. 2016) and some efforts are underway to help characterize the clinical correlates of this pathology; however, AMS-TBI is inexplicably excluded from currently proposed case definitions of traumatic encephalopathy syndrome (Montenigro et al. 2014; Reams et al. 2016). As such, the community of AMS-TBI researchers is charged with: 1) establishing a shared nomenclature and operational definition of post-traumatic dementia in AMS-TBI, and 2) common methods and data sharing approaches specific to AMS-TBI so that imaging can be leveraged to advance discovery. Given this background, early efforts for the AMS-TBI working group will be to support data harmonization, analyses and, ultimately, prospective data collection for detailed analysis of mechanism for neurodegenerative risk in msTBI. Novel analysis techniques may also help to interpret longitudinal changes as well as help predict ongoing trajectories of change. For example, there is a literature using functional MRI (Crone et al. 2018; Hillary et al. 2014; Rajtmajer et al. 2015; Roy et al. 2016), structural (Lutkenhoff et al. 2013, 2019), and multimodal data (e.g., structural MRI and EEG; Schnakers et al. 2019) to examine recovery in small samples of AMS-TBI during the first year post injury, but we also need more robust methods tailored for more long-term consequences of TBI (Cruz-Haces et al. 2017) which can also handle heterogeneous outcome trajectories. We anticipate that international collaborations, with careful meta-analysis of data from multiple centers, will provide novel avenues for exploring and clarifying chronic and long-term effects of TBI.

### Integration of MRI data with other imaging techniques and non-imaging biomarkers

While the focus of ENIGMA AMS-TBI is on imaging and particularly MRI methods, there is now substantial evidence that no existing single imaging modality or diagnostic /neuromonitoring tool is sufficient for characterization and phenotyping in TBI (Amyot et al. 2015; Mondello et al. 2018a). The logical next step is to identify a multidimensional profile employing distinct classes of emerging technologies that convey diverse, complementary and independent information thereby enabling clinicians to achieve better characterization of patients with TBI and stratify risk more effectively. Such strategy is likely to provide a greatly expanded understanding of the pathogenesis and consequences of TBI, making it possible to transform health care delivery and improve patient outcomes by individualizing management and intervention. Future directions of our working group relying on the synergy of multidisciplinary collaboration will therefore entail focus on the development of novel methods for robust integration of different advanced neuromonitoring tools in our large-scale imaging analyses. Current participating sites have datasets including *PET imaging, MRS, EEG*, as well as *fluid biomarkers*.

*PET imaging* has been used to assess early injury mechanisms (Bergsneider et al. 2001; Coles et al. 2004), recovery (Yamaki et al. 2018), long-term neural consequence (Barrio et al. 2015; Bodart et al. 2017; Lupi et al. 2011) and neural correlates of functional deficits (Buchsbaum et al. 2015; García-Panach et al. 2011; Komura et al. 2019; Nakashima et al. 2007; Spadoni et al. 2015) or interventions (Östberg et al. 2018; Scott et al. 2018). PET is a valuable approach for methodological corroboration of structure and functional brain results and can elucidate TBI pathophysiology in a manner not possible with MRI or CT. For example, one current hypothesis regarding the role of increased connectivity following TBI (i.e., hyperconnectivity) is that while potentially “compensatory” for function, enhancement of functional connections has longer term metabolic costs resulting in pathological protein aggregation (Hillary and Grafman 2017). With metabolic imaging this hypothesis can be evaluated directly. Tau deposition has more recently been observed in mixed TBI samples (see Gorgoraptis et al. 2019; Wooten et al. 2019). In the work by Wooten and colleagues, these findings where spatially co-localized with sites showing the greatest network connectivity established using fMRI. [C-11] PiB PET has also demonstrated amyloid aggregation in msTBI (Hong et al. 2014; Scott et al. 2016), recapitulating the temporal pattern seen in post mortem findings, and demonstrating specific early striatal deposition not detected by autopsy studies. Finally, there are now tracers targeting activated microglia (Coughlin et al. 2015; Scott et al. 2018) which permit examination of the shifting balance between pro- and anti-inflammatory processes induced by microglial activation that allow for restoration rather than perpetuation of injury (Sandvig et al. 2018) and thus potentially improve TBI outcome. Combining PET and MRI data has a great potential to advance the understanding of the primary and secondary pathophysiological mechanisms in TBI and future efforts in our working group will be focused on this vital integration of data types.

*Magnetic resonance spectroscopy (MRS)* is (unlike PET) a noninvasive tool to measure brain metabolites that are indicative of injury. MRS has shown alterations in metabolites reflecting neuronal health and cellular turnover in TBI (Bartnik-Olson et al. 2019; Brown et al. 2018). Whole brain N-acetylaspartate (NAA), which is a proxy for neuronal integrity, and choline (Cho), a cell membrane marker, correlate with injury severity and neuropsychological functioning (Govind et al. 2010; Govindaraju et al. 2004; Maudsley et al. 2015), as well as with coarse global outcomes (GOS) at 3 months post injury (Marino et al. 2007). Across all severity ranges, the predictive utility of MRS for long-term functioning is above and beyond other clinical indicators and conventional structural imaging findings. Since our working group will include several additional imaging modalities, the unique contribution of MRS to help elucidate the pathobiology imaged by these other modalities (structural and functional) can be leveraged further.

*Electroencephalography (EEG)* is a non-invasive technique that can provide valuable information about sensory and higher order cognitive processing after TBI. Sensory evoked potentials can provide vital information related to the integrity and functionality of peripheral pathways and spinal tracts (Carter and Butt 2005; Munjal et al. 2010). Other uses of EEG include the assessment of the level of consciousness in patients with AMS-TBI who lack behavioral evidence of language expression and comprehension (Braiman et al. 2018; Cruse et al. 2011; Edlow et al. 2017). The power and power variability of different frequency bands at an early stage after injury (1–10 days in most studies) has been linked to global outcome (typically GOSE 6 months after injury; see Hebb et al. 2007; Schnakers et al. 2019; Tolonen et al. 2018; Vespa et al. 2002). Moreover, the amplitude and latency of event related potentials (ERPs) can support inferences about the nature of specific cognitive impairments (e.g. processing speed, sustained attention, performance monitoring and inhibitory control; see reviews by (Dockree and Robertson 2011; Duncan et al. 2005; Folmer et al. 2011). Currently, there are few studies in AMS-TBI combining EEG and advanced neuroimaging techniques. One recent example of a study combining EEG with MRI and DTI demonstrated a link between higher neuronal synchrony during sleep and white matter damage in frontal and temporal brain regions (Sanchez et al. 2019). Our working group will collaborate with the ENIGMA-EEG working group to leverage the full potential of combining EEG data with other imaging techniques in msTBI.

*Fluid biomarkers* have emerged as an objective and powerful tool for aiding clinical diagnosis, monitoring the progression of damage or response to treatment, serving as surrogates of clinical outcomes and characterizing pathogenic mechanisms and potential therapeutic targets in patients with TBI (Mondello et al. 2018b; Undén et al. 2013). Such potential has dramatically stimulated the quest for new markers and accelerated their integration into clinical decision rules (Mondello et al. 2018b; Undén et al. 2013). Moreover, to better account for the complexity of the molecular, biologic and pathologic events triggered by the injury, an intense effort is underway to expand biomarker role and integrate them into novel sophisticated approaches of multidimensional classification of TBI (Ko et al. 2019; Nielson et al. 2017; Thelin et al. 2017).

Examination of advanced MRI data in relation to glial and neuronal markers in blood has shown that acute assessment of glial fibrillary acidic protein [GFAP] correlates closely with intracranial bleeding as assessed by susceptibility weighted imaging (SWI) but not with other imaging techniques (Kou et al. 2013). More recently, extending and corroborating this research, Gorgoraptis and colleagues (Gorgoraptis et al. 2019) have reported in individuals who have suffered a moderate to severe TBI an association between PET data, MRI correlates and CSF markers of neurodegeneration (Tau and ubiquitin carboxy-terminal hydrolase L1 [UCH-L1]) many years after the injury, concluding that increased flortaucipir (i.e. a radioligand for tau) binding is indicative of the presence of tau pathology and traumatic axonal injury which are, in turn, interconnected. Taken together, these observations emphasize the usefulness of comparative analysis between fluid biomarker and neuroimaging to solve interpretative problems and comprehensively understand the molecular mechanisms and pathogenesis of the observed damage, tying different features (quantification, distribution, kinetics), and neuropathological conditions (e.g. tau deposition, macromorphological and micro-structural changes of white and grey matter) to underlying biomarkers profiles and disease processes (e.g. neuronal loss, axonal injury and progressive neurodegeneration). It is, therefore, tempting to speculate that this approach creates the opportunity for identifying specific -possibly unique- multimodal signatures of TBI and represents a “new paradigm of hypothesis-generating research”.

Despite the recently developed sensitive and high-sensitivity assays that have been shown to improve the diagnostic accuracy of TBI complementing neuroimaging techniques, to date, operational strategies to best take advantage of these tests in clinical practice are limited. Two clinical studies (Gill et al. 2018; Yue et al. 2019) by independent groups have demonstrated in large cohorts that analysis of blood GFAP concentrations can identify patients with TBI who might need subsequent MRI examination. These findings pave the way for the incorporation of GFAP into clinical algorithms for diagnostic workup strategy selection. Furthermore, combining fluid and imaging biomarkers may also turn into valuable means to understand treatment mechanisms and evaluate eventual therapeutic interventions. To this end, a recent study has shown that the combination of PET, MRI and NFL provides unique information for better understanding the effects of minocycline on microglial activation (Scott et al. 2018).

Despite these promising examples, this work is still in its infancy. Further validation, standardization and evidence of clinical utility beyond current practice standards is needed. In this regard, ENIGMA AMS-TBI can contribute importantly to this growing base of evidence by 1) identifying and unveiling the relationships between advanced imaging techniques, fluid biomarker and clinical outcomes, 2) demonstrating and validating the added value (e.g. improved damage characterization, quantification) when combining these different tools, and 3) determining the context in which the conclusions apply (i.e. clinical utility for a specific contextual use). ENIGMA AMS-TBI is also uniquely suited for playing an instrumental role in developing and defining clinical algorithms and recommendations/guidelines which identify the best practices integrating biofluid and imaging markers for the diagnosis, prognosis, and management/treatment of patients with msTBI. Finally, if successfully implemented, this research framework could be adopted as a blueprint across other neurological and neurodegenerative disorders.

### Harnessing the power of radiogenomics in msTBI

Genetic factors modulate host-response and secondary injury after TBI, and may therefore account for some of the unexplained variation in outcome (Maas et al. 2017). Much existing literature has focused on the impact of genetics in mild injury (McFadyen et al. 2019; Nielson et al. 2017; Winkler et al. 2017) or on global clinical outcome (Conley et al. 2014; Dardiotis et al. 2014; Failla et al. 2015a). However, (other) reports in msTBI have explored the relationships between genetics (particularly single nucleotide polymorphisms) and more granular pathophysiology-based outcomes such as cognition (Ariza et al. 2006; Failla et al. 2015b; Isoniemi et al. 2006; Koponen et al. 2004; Krueger et al. 2011; Markos et al. 2017; McFadyen et al. 2019; Nicoll et al. 1995; Wagner et al. 2012), seizures (Darrah et al. 2013; Diamond et al. 2015; Diaz-Arrastia et al. 2003; Ritter et al. 2016; Wagner et al. 2010; Wang et al. 2017), cerebral autoregulation and blood flow (Chen et al. 2011; Robertson et al. 2011), cerebral edema (Jha et al. 2019, 2018, 2017), and contusion expansion (Hadjigeorgiou et al. 2005; Liaquat et al. 2002). While many of these genetic association analyses have used neuroimaging metrics, the more common use of structural and functional neuroimaging as endophenotypes of outcome (Coughlin et al. 2017, 2015; De Simoni et al. 2016; Hong et al. 2014; Lutkenhoff et al. 2015; Newcombe et al. 2016, 2013; Schnakers et al. 2019; Scott et al. 2016; Stender et al. 2014; Veenith et al. 2014; Zheng et al. 2017) have been less well studied in the context of studies evaluating the genetic influence on disease process after msTBI. Global TBI outcomes are complex and multifaceted, and the role of genetic variation on eventual outcome, months to years after injury, may be confounded by multiple intrinsic and extrinsic factors. These difficulties may be mitigated by better understanding of the molecular mechanisms that underpin secondary injury, and identifying candidate imaging phenotypes which can be rationally related to these processes. Thus, genetic determinants of cerebral edema, late neurodegeneration, or neuroplasticity (all of which have imaging phenotypes that are discussed elsewhere in this manuscript) may differ from one another in effect (identity and/or direction). These more specific outcomes may show detectable genetic associations which are diluted or undetectable in studies evaluating global clinical outcome.

Clinical radiogenomics (the correlation of genetic signatures with imaging) was pioneered in tumor biology research, where it has informed treatment and prognostication (for review see Pinker et al. 2018). Although less commonly used in TBI, this approach has been applied to imaging phenotypes in TBI, (Table 1) leveraging knowledge of underlying molecular mechanisms to direct investigation of specific genes/pathways. However, such studies are limited by small sample sizes which lack the statistical power to detect rare (but mechanistically important) genetic variations, or to validate them in unbiased genome-wide association studies (GWAS). Although multicenter consortia (such as CENTER-TBI and TRACK-TBI) are beginning to deliver the sample sizes needed, radiogenomic research in msTBI is challenged by the time and cost of acquiring imaging datasets, differences in image acquisition across centers, failure of conventional image processing tools in severely injured brains, and variations in data processing and access to genetic data across research groups. A collaborative, standardized and accessible platform for researchers to process, integrate, harmonize, optimize, analyze and disseminate imaging phenotypes is thus imperative to advance fundamental knowledge about the impact of genetics on imaging metrics of disease biology and outcome after TBI.

**Table 1:** Summary of studies in TBI correlating genetic profiles with imaging phenotypes

Gene Name	Rationale	Outcome Measure	Sample Size	Single Center	Results	Reference
*CACNA1A*	Association of familial hemiplegic migraine attacks and coma with minor head trauma, mechanistically linked to calcium channel mediated glutamate release	CT EEG	3 (152 control) 2	Y Y	Missense S218L present in all subjects with delayed malignant cerebral edema and absent in 152 controls and unaffected family members De novo S218L mutation in both subjects with seizures and severe cerebral edema	Kors et al. (2001) Stam et al. (2009)
*APOE-ε4*	Previous association reports with unfavorable outcome after TBI	Hematoma Volume (CT)	129	Y	Larger hematomas in carriers of *APOE-ε4* allele	Liaquat et al. (2002)
	Neurodegenerative process accelerated after blast injury; increased risk of APOE-e4 carriers to develop Alzheimer’s Disease after TBI	White matter integrity (MRI DTI)	217	Y	Interaction between close-range blast exposure and *APOE ε4* carrier status in predicting white matter disruption	Sullivan et al. (2019)
*IL-1RN* *IL-1B*	IL1-RN and IL-1B (inflammatory markers) are elevated post-trauma and implicated in blood vessel wall stability	Hematoma volume (CT)	151	Y	Association between IL-1RN*2 allele status and hemorrhage	Hadjigeorgiou et al. (2005)
*NOS3*	Established pathophysiologic role of endothelial nitric oxide synthase in maintenance of cerebral blood flow	Xenon CT Transcranial doppler	51	Y	Cerebral hemodynamics related to -786 T > C genotype	Robertson et al. (2011)
*DAT* *DRD2*	Dopamine transporter (DAT) binding reductions after severe TBI, role in cognition.	PET	25	Y	*DAT* 9-allele carrier and *DRD2* A2/A2 homozygotes showed lower caudate and putamen binding	Wagner et al. (2014)
*ABCC8* *TRPM4*	Sulfonylurea-receptor 1 (encoded by ABCC8) association with TRPM4 after brain injury creates a pore-forming channel facilitating depolarization and cerebral edema	CT ICP	385–410	Y	Regionally clustered *ABCC8* and *TRPM4* polymorphisms were associated with CT edema, intracranial hypertension and outcome. Significant interactions noted between predictive variants in the 2 genes.	Jha et al. (2017) Jha et al. (2018) Jha et al. (2019)

**Fig. 5:**
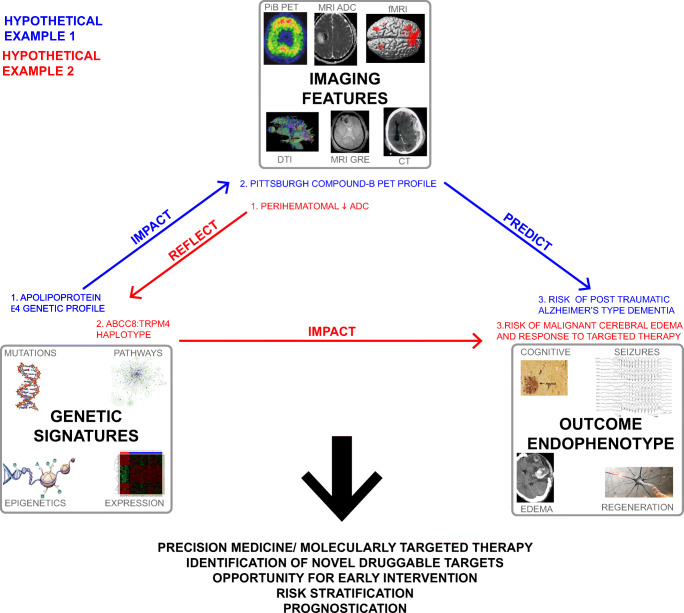
Future vision of radiogenomics in moderate/severe TBI. This schematic demonstrates relationships between genetic signatures, imaging characteristics, and endophenotype outcomes after msTBI. Genetic signatures can be in the form of mutations, regulation, expression profiles, and epigenetics for single-genes or pathways. Different genetic signatures impact specific outcome phenotypes (for example: neurodegeneration/cognition, seizures, cerebral edema, neural regeneration). Some of these outcome endophenotypes may be detected acutely (e.g. cerebral edema), whereas others may have a temporal lag varying from days-years until clinical detection (seizures, neurodegeneration). Imaging features may serve as surrogates for certain outcome endophenotypes- for example, MRI based ADC hypointensity may reflect cytotoxic edema, or PiB detection of amyloid aggregation may portend risk for Alzheimer’s Disease. Genetic signatures can thus be linked to imaging features as proxies for an endophenotypic outcome, or interpreted synergistically. Hypothetical example 1 (blue) suggests that a specific *APOE e4* genotype (blue-1, genetic signature) results in a certain PiB-PET profile (blue-2, imaging feature); these two features combined may predict risk of post-traumatic Alzheimer’s type dementia (blue-3, endophenotype outcome). Hypothetical example 2 (red) indicates that detection of perihematomal ADC reduction (red-1, imaging feature) reflects a specific ABCC8:TRPM4 haplotype (red-2, genetic signature); this haplotype impacts risk of malignant cerebral edema and mediates response to therapy. (Of note, ABCC8 and TRPM4 encode subunits of an octameric cation channel known to mediate cerebral edema after brain injury). Identifying the relationship between genetic signatures, imaging features, and outcome endophenotypes for different secondary injury processes will facilitate precision medicine, identification of novel targets, opportunities for early intervention, risk stratification and prognostication, ABCC8 = ATP binding cassette subfamily C member-8; ADC = apparent diffusion coefficient; APOE-e4 = apolipoprotein E epsilon 4; MRI = magnetic resonance imaging; msTBI = moderate-severe TBI; PET = positron emission tomography, PiB = Pittsburgh compound B, TRPM4 = transient receptor potential cation channel subfamily M.

ENIGMA will provide such a framework to leverage imaging and genomic data obtained from existing multicenter initiatives, single center datasets, and future studies. This will facilitate research evaluating effects of genetic variation (mutations, gene expression, epigenetics, pathway analyses) on distinct pathophysiological processes, using imaging metrics as phenotypes for association. This could deliver better understanding of disease biology and identify new druggable molecular targets. Combining genetic profiles and imaging features also has powerful synergistic potential in terms of risk stratification and precision medicine, providing opportunities for early, targeted intervention and prognostication (Fig. 5). For example, despite extensive study of *APOE-e4* genotypes in TBI, its precise relationship with modulating outcomes is undefined (for review see McFadyen et al. 2019). Complementing genotype data with serial PiB PET imaging characteristics may provide pathophysiologic insight and improve prediction of risk for post-traumatic Alzheimer’s Disease (Fig. 5).

## Conclusion

The ENIGMA AMS-TBI working group was recently initiated to address an unmet need for a robust framework to leverage imaging data in AMS-TBI research. Here we have outlined the background and structure of ENIGMA AMS-TBI, roles of investigators and our *short, intermediate and long-term goals*. The approaches and initiatives within the group will be shaped by the joint efforts of existing and future new members. We encourage researchers who are interested in contributing to this work to contact the authors of this paper to join our efforts toward a global and reproducible science for brain imaging in neurotrauma.

## Footnotes

1 Adult in this context is broadly defined as >16 years old.
